# Patients’ experiences of clinical trial participation involving a product remotely assessing study drug adherence

**DOI:** 10.1016/j.conctc.2024.101307

**Published:** 2024-05-13

**Authors:** Catrin Henriksson, Anneli Olsson, Kasper Andersen, Gabriel Arefalk, David Erlinge, Robin Hofmann, Wilhelm Ridderstråle, Annika Rutgersson, Jonas Oldgren, Stefan James

**Affiliations:** a Cardiology, Department of Medical Sciences, Uppsala University, Uppsala, Sweden; b Cardiology, Department of Clinical Sciences, Lund University, Lund, Sweden; c Thoracic Centre, Blekinge Hospital, Karlskrona, Sweden; d Division of Cardiology, Department of Clinical Science and Education, Karolinska Institutet, Södersjukhuset, Stockholm, Sweden; e Late-Stage Development, Cardiovascular, Renal and Metabolic, Biopharmaceuticals Research & Development, AstraZeneca, Gothenburg, Sweden; f Digital Health, Biopharmaceuticals Research & Development, AstraZeneca, Gothenburg, Sweden; g Uppsala Clinical Research Center, Uppsala University, Uppsala, Sweden

**Keywords:** Patient experience, Myocardial infarction, Trial participation, Medical technology

## Abstract

**Background:**

The participation of patients in clinical trials is crucial for the development of healthcare. There are several challenges in the recruitment of trial participants with acute medical conditions. The registry-based randomized DAPA-MI clinical trial recruited patients during hospitalization for myocardial infarction and provided study drugs in bottles with smart caps that used wireless technology to transmit monitoring data. This interview study aimed to investigate patients’ experience of participation in a clinical trial and their attitude to the new bottle cap technology.

**Methods:**

A subset of patients participating in the DAPA-MI trial were recruited from four hospitals in Sweden. Semi-structured interviews were conducted and analysed using manifest content analysis.

**Results:**

Video interviews were performed including 21 patients (four women and 17 men). The median age was 59 years (range 44–80). Four categories of patients' experiences were identified. *A willingness to contribute* consisted of patients’ positive attitudes to participation and to be a part of development and research. *The perception of information* emphasized the value of the oral information as well as the importance of time for reflection. *Be in a vulnerable condition* highlighted the impaired ability to perceive and remember in the acute medical condition. *Adaptation to a new technology* described the overall positive experiences of the smart bottle cap to evaluate adherence.

**Conclusions:**

Patients’ experiences of trial participation were in general positive but some challenges in the acute setting of a myocardial infarction were revealed. The smart bottle cap was well accepted, despite some handling difficulties.

## 1 Introduction

Trial participation is fundamental to research progress and development of new treatments, strategies, and technology. This requires voluntary and dedicated participants. However, recruitment of participants who are as similar as possible to the overall population, as well as retention, is challenging. It can be difficult in many randomized controlled trials to achieve the targeted sample size within the planned time frame [1]. Patient and public involvement in trial design have been beneficial to recruitment and retention [2], and use of valid patient experience data is the basis to evaluate existing research designs and different approaches for patient recruitment.

Several factors can influence a person's decision to take part in a trial, such as the potential benefits to the participant and the time commitment required, as well as their confidence in the physician's and research nurse's expertise [[3], [4], [5]]. Recruiting for clinical trials in acute conditions, such as myocardial infarction, poses additional challenges because of time limitations, stress, and severe illness. Moreover, the willingness to participate under such circumstances also depends on the type of the trial [6]. Using existing clinical registries embedded in healthcare to facilitate the conduct of a randomized clinical trial is a possible strategy to recruit a broad population representative of clinical reality [7]. In addition to its registry-based approach, the DAPA-MI trial (Dapagliflozin in Patients without Diabetes Mellitus with Acute Myocardial Infarction; NCT04564742) [8] used a smart bottle cap affixed to study drug bottles to monitor study drug adherence. This innovative way of conducting trials and monitoring study drug adherence needs to be further evaluated. While a registry-based approach can reduce the burden of carrying out clinical trials on healthcare systems and facilitate fast recruitment and efficient data collection, the patient experience of participation in such a trial will be important to improve future research. Indeed, a review published in 2021 highlighted the need for more research into clinical trial participant perspectives and experience across the spectrum of clinical trials [9]. A better understanding of patient perspectives will help to inform the design of future clinical trials, and an understanding of their experiences using new technology to aid medication adherence could also help to inform strategies for improving adherence in routine clinical practice.

The aim of this study was therefore to attain a better knowledge and understanding of patients’ experiences of participation in the registry-based randomized clinical trial DAPA-MI and to evaluate their attitude towards the smart bottle caps used to monitor study drug adherence.

## 2 Method

This was an interview study with some individuals participating in the DAPA-MI trial [10]. The study had a qualitative design and included individual semi-structured interviews by video.

### 2.1 DAPA-MI trial

**Fig. 1:**
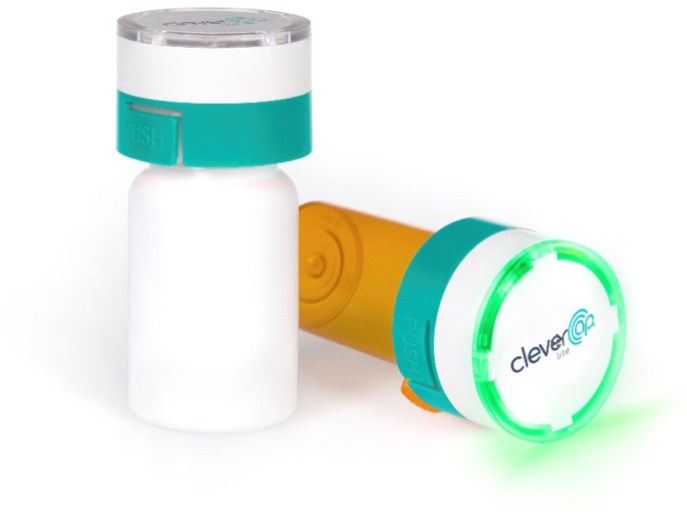
The smart bottle cap with remote monitoring function (CleverCap Lite), used in the DAPA-MI trial to evaluate study drug adherence. Picture published with permission.

The DAPA-MI trial was a registry-based, double-blinded, placebo-controlled, randomized clinical trial evaluating the utility of dapagliflozin early after acute myocardial infarction in patients with impaired left ventricular systolic function but without diabetes [8]. The protocol allowed inclusion and randomization within 10 days after admission for myocardial infarction (index hospitalization). The first follow-up visit occurred from 6 to 10 weeks after randomization, and the following visits were planned every 10 months. The DAPA-MI trial used a smart cap affixed to study drug bottles which used wireless technology (CleverCap Lite; Compliance Meds Technologies, Ives Estates, FL, USA) to monitor drug adherence (Fig. 1). When the cap was opened, a wireless signal was sent to a central portal using the 3G network. This real-time monitoring allowed for less-frequent trial visits for the participating individuals. If unexpected user patterns were detected, an automated e-mail notification was sent to research nurses to follow up with the patient. The cap had an integrated audio notification system that played a sound to inform patients when the cap was opened and could also alert them to low battery status.

### 2.2 The SWEDEHEART registry

The SWEDEHEART registry includes all patients admitted to coronary care units with confirmed myocardial infarction, and all hospitals in Sweden providing care for patients with myocardial infarction participate in the registry collaboration [11]. A follow-up visit is scheduled at 6–10 weeks after discharge and again after 12 months for patients up to the age of 80 years. This routine care follow-up schedule was used for Swedish patients in the DAPA-MI trial.

### 2.3 Population

The interview study population consisted of a subset of participants included in the DAPA-MI trial. Inclusion criteria for participating in the interview study were access and ability to use a web-based communication platform. Exclusion criteria specific for this study included language difficulties and confusion. Ten Swedish hospitals were asked to participate, and four hospitals accepted the invitation.

### 2.4 Procedure

At the follow-up visit, the research nurse at each hospital asked every second patient if they were interested in participating in the interview study. The patients received oral and written information, and if the patient accepted further information, the research nurse provided the researcher (first author) with the individual's name and telephone number. Thereafter, the patients were called by the researcher and received more detailed, verbal information. The researcher asked for the patient's e-mail address and additional written information was sent. Subsequently, each participant was asked to suggest dates for the interview, and finally one date was decided.

**Fig. 2:**
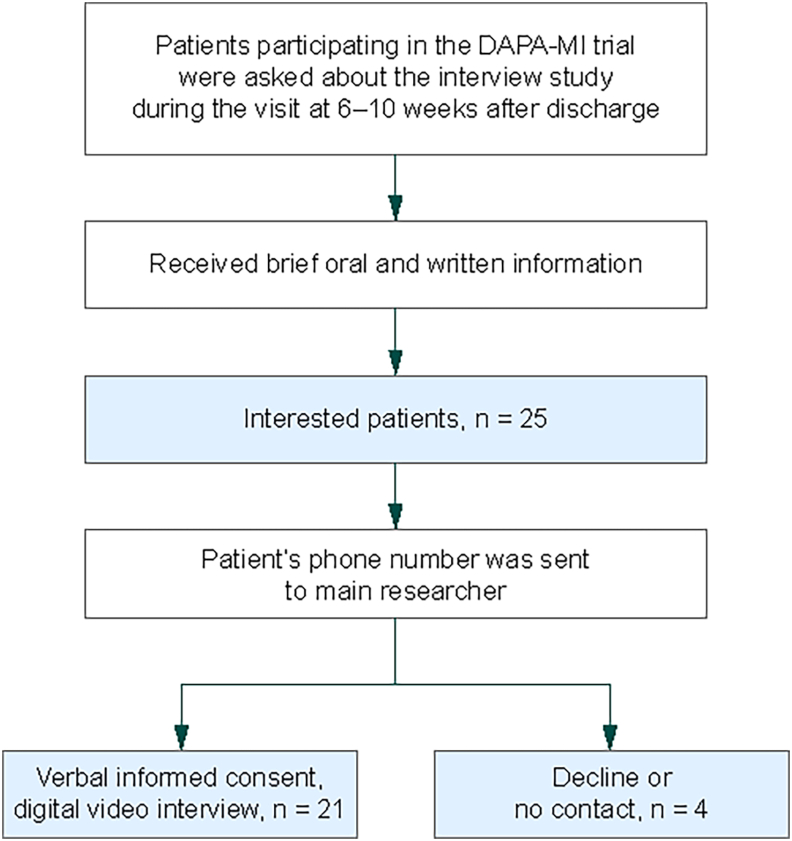
Patient identification flowchart for the interview study.

The persons who agreed to the interview gave their oral consent to participate in the study when the interview started. The interviews were performed by a web-based communication platform and lasted in total 15–20 min. The researcher (CH) stopped the recruitment when no new substantive information was acquired, and a variation of participants had been interviewed in relation to age and sex. The trial design is visualized in Fig. 2.

The main questions in the semi-structured interview were as follows.

• How did you experience being asked to participate in a clinical trial?
• How did you experience the information about the DAPA-MI trial?
• What is your experience using the bottle cap?
• What is your experience with the technology around the bottle cap?

Every interview was recorded and thereafter transcribed by the responsible researcher. After transcription of the material, the text was analysed according to manifest, qualitative content analysis [12]. Words and sentences containing information relevant to the aim of this study were identified and classified as meaning units, which were then condensed. The condensed meaning units were grouped to form subcategories and were finally deployed to build main categories (Table 1). The data were collected and analysed by the same person (CH). The coding and categorization were assessed by the second author (AO) in discussion with CH.

**Table 1:** Examples of meaning units, condensed meaning units, codes, and categories.

Meaning unit	Condensed meaning unit	Code	Sub-category	Category
#14, male, 55 y; *I thought about decline, but it felt good to be able to help*	… felt good to be able to help …	Good to help	Willingness	A willingness to contribute
#4, male, 47 y; *Interesting to be involved in developing something that can be of social benefit*	… involved in development … can be of social benefit …	Involved potential benefit	Contribution to research	
#19, male, 58 y; *I think it was very good with both verbal and written information*	… good with both verbal and written information …	Verbal and written	Content of information	The perception of information
#1, male, 56 y; *There is a risk of over-information, which is confusing*	… over-information gives confusion …	Confusing if too much information	Understanding of information	
#13, female, 73 y; *I had so many other things to think about I don't know how aware I was*	… other things in mind …… not aware …	Distracted by other things	Lack of awareness	Be in a vulnerable condition
#18, male, 49 y; *I don't remember exactly, but I have it (the information) on a paper*	… don't remember exactly …	Remembrance	Insufficient remembrance	
#1, male, 56 y; *I felt it was exciting with a new technical product*	… exciting with a new technical product …	Excitement	Interest in technology	Adaptation to a new technology
#7, male, 59 y; *It was a little bit tricky to open at first*	… tricky to open …	Handling difficulties	Technical issues	

### 2.5 Ethics

The interview study was approved by the Swedish Ethical Review Authority (Dnr 2021–02963). The investigation conformed to the principles outlined in the Declaration of Helsinki.

## 3 Results

Of the 10 Swedish hospitals that were asked to participate, four geographically spread hospitals recruited individuals to this study. The study population consisted of 21 persons (four women and 17 men). The median age was 59 years (range 44–80 years). The patients were recruited between September 2021 and April 2022.

Four categories of patients’ experiences were identified in the semi-structured interviews (Table 1).

*A willingness to contribute* consisted of patients’ positive attitudes to participation and to be a part of development and research. *The perception of information* emphasized the value of the oral information as well as the importance of time for reflection. *Be in a vulnerable condition* highlighted the impaired ability to perceive and remember in the acute medical condition. *Adaptation to a new technology* described the overall positive experiences of the smart bottle cap with remote monitoring function to evaluate study drug adherence.

### 3.1 A willingness to contribute

Most participants described positive experiences to participate in a clinical trial and felt a willingness to help. Several patients described their gratefulness for being able to help with the development of medical care: one patient said “I thought about declining, but it felt good to be able to help” (#14, male, 55 y). Some participants commented that the possibility of being a part of public welfare and the development in science were of special interest: “… interesting to be involved in developing something that can be of social benefit” (#4, male, 47 y), and “I'm positively curious to participate in the (DAPA-MI) study. There is something going on to improve, a chance to get healthier” (#1, male, 56 y). Another participant commented that participation was “good for the future …-a chance to influence” (#4, male, 47 y).

Some of the participants described feelings of being specifically selected: “It was a privilege to be asked [to participate], relevant information and what [participation] could lead to” (#3, female, 60 y). No patients expressed a feeling of dependence on the physician who provided the trial information, and one participant said: “He (the physician) was in position of dependence to me” (#2, male, 69 y).

### 3.2 The perception of information

The verbal and written trial information was mainly perceived as sufficient: “The information was good, I could ask the questions I wanted” (#2, male, 69 y). Some feedback was that it included too much information: one person stated that “there is a risk of over-information, which is confusing” (#1, male, 56 y). Some participants thought that the information should not be given early during the hospitalization: “I received the information a little early, it would have been better if I had received the information closer to discharge” (#13, female, 73 y). Another patient, on the other hand, appreciated that he was given time to consider his decision: “The possibility of time for consideration was positive” (#20, male, 69 y).

The provision of verbal information about the study was perceived as being particularly meaningful, although the combination of both verbal and written information was also appreciated: one patient stated “I think it was very good with both verbal and written information” (#19, male, 58 y), and another patient thought that “The oral information was most important” (#4, male, 47 y). How the information was perceived depended on the person who gave the information: one person described his experience: “The one who gave the information didn't look anxious, she looked confident and gave trust” (#1, male, 56 y). Another aspect was that some of the participants appreciated if the person providing the information was not directly involved in the care: “The person who informed me was not the care-giving personal, it was very clear and an advantage” (#4, male, 47 y) and “It was perceived as advantageous for the information to be given by trial staff not involved in medical care” (#1, male, 56 y).

### 3.3 Be in a vulnerable condition

Given that the patients were undergoing acute care for myocardial infarction at the time of receiving information on the trial, it is perhaps unsurprising that several persons mentioned that they did not remember a lot of the information. Some reflections consisted of: “I had so many other things to think about, I don't know how aware I was. I had not really understood that I had a myocardial infarction” (#13, female, 73 y) and “[It was] hard to process everything. Didn't know what I really signed, but the physician said that I could withdraw my approval at any time” (#15, female, 44 y). One male patient, aged 49 years (#18), described the information as “… rather good. I don't remember exactly, but I have it (the information) on a paper”, further highlighting the importance of the provision of both verbal and written information.

The possible risk of participation was something that a few participants reflected on. One patient commented: “I learned the purpose of the trial, it was an approved substance. I think it was good, a minor risk” (#7, male, 59 y). The perception of low risk for participating in a clinical trial of an approved substance may have also influenced patients' willingness to help despite their medical condition. The possibility of more direct access to healthcare was also described: “Easier to get in touch with healthcare” (#12, male, 51 y). This reflected patients’ desires to feel safe when receiving treatment for their condition, as well as their appreciation for continuous contact with dedicated research staff.

### 3.4 Adaptation to a new technology

Most experiences of the smart bottle cap were positive and gave no negative feelings of control:. “I felt it was exciting with a new technical product” (#1, male, 56 y), and “Smart solution, no problems. I have no feelings of being controlled, this function is for my best” (#10, male 48 y). Participants believed that it was an advanced cap with a clear signal when it had to be charged. At the time of the interviews, no one had so far been contacted by the research nurse regarding insufficient adherence. One person wished that all medications should include an alarm as a control function to ensure that the medication was taken. “It would be an advantage if it (the smart bottle cap) had an alert signal if I haven't taken my pills” (#6, female, 65 y). Some patients had previously contacted the research nurse about using a 7-days pill organizer. “The ability to use the smart bottle cap once a week was an advantage” (#13, female, 73 y). This highlights the importance of giving patients the opportunity to adapt their care to a format that is more convenient for them.

A few participants experienced some challenges with the smart bottle cap. The most common problem was that they either forgot or did not receive information that the cap needed regularly charging. Some participants experienced practical problems with the cap, and one man expressed: “I have poor vision and could not see that I should press on the cap to open it” (#7, male, 59 y).

## 4 Discussion

To our knowledge, the DAPA-MI trial is the first trial that used both a registry embedded in healthcare and a technological product to improve adherence to the study drug. Our findings in this interview study show that participants’ experiences of taking part in a registry-based clinical trial were generally positive, with participants often exhibiting a willingness to help. The provision of verbal information during the trial was considered most useful. Some of the participants did not remember the content of the trial information but expressed great confidence in the research staff. The smart bottle cap was well accepted and gave no negative feelings of being controlled.

In the current study, the population was young compared with the general population of patients with myocardial infarction and consisted primarily of men. This may have influenced their positive attitude to technological products; however, other studies have also found similar positive results [13,14].

One important reason for participation agreement seems to be the willingness to help in general. This phenomenon was seen both in our study and in a previous study [13]. The person's gratefulness for medical care and being alive after an acute myocardial infarction might influence the decision. In addition, feelings of being chosen and being a part of science and public welfare were experienced positively.

Somewhat surprisingly, none of the participants experienced any feelings of dependence in relation to the physician during the decision process. This is in line with a previous study investigating a population admitted with ST-elevation myocardial infarction which found that only 2 % of participants experienced any feeling of pressure [14]. Several participants in the current study experienced the informer as trustworthy and confident, which could have influenced their perception positively. It was also perceived as an advantage if the trial physician was not involved in the routine medical care, such that a more trusting relationship between the treating physician and the patient was more likely if no research was discussed.

How much the information was remembered by the participants differed among individuals. It is a known problem that patients with myocardial infarction in acute settings have a limited ability to remember extensive information [3,15]. It has been previously shown that 20 % of participants admitted with acute myocardial infarction reported that they did not know in which clinical trial they were participating [14]. There is a risk that the participant does not understand the information or denies the knowledge as a result of feeling shocked at having received a cardiac diagnosis.

Some participants preferred to be asked to participate later during the hospitalization, and some commented that there were so many other things to think about and even that they did not even understand that they had a myocardial infarction. To reduce such problems, it is important to individualize the information, take into account how a patient may reflect on the situation, and facilitate the partnership with the patient in accordance with person-centred care [16].

The potential risk associated with participation in a clinical trial were only noticed by a few persons. This finding was strengthened by a study showing that the participants agreed to the statement that “only safe drugs are tested in human research” [17]. If the study drug was an approved substance, such as in the current study, it will contribute positively to the participants’ decision.

Surprisingly, no participant believed the smart bottle cap had a negative control function. On the contrary, it was perceived as a reminder and a function in their best interest. Some participants noted problems opening the bottle, but the experiences were mainly positive. It is important to consider ease of use and eventual problems with muscular weakness and visual impairment in future development of such devices. The advantage of this technology is the real-time monitoring of study drug adherence and an opportunity to follow up with patients that have patterns of irregular or low adherence.

### 4.1 Strengths and limitations

The study used a qualitative design with relatively few participants and may therefore not be generalizable, but it provides some information of the patients’ thoughts and experiences. Some advantages in this study were that the data were collected and analysed by the same person. To further ensure the credibility of the data analysis, the content of each main category was examined in collaboration with the second author.

The interviews lasted for 15–20 min. This short duration was chosen to minimize disruption to patients and because of the virtual nature of the interviews. Since both body language and gestures are harder to interpret in a virtual meeting (for both the researcher and the interviewed patient), this may have led to some missing information compared with physical meetings. In-person focus group interviews were considered as an alternative, but because the interviews were performed during the COVID-19 pandemic, this was ruled out.

The four hospitals asked every second patient to participate in the study. It was decided to sample patients in this way to attempt to recruit study participants with variation in key demographics (e.g. age, sex, and geographical area) similar to the DAPA-MI trial itself, and to avoid any bias that selective sampling may introduce. This method for selecting patients also helped to facilitate recruitment because the researcher was unable to travel to each hospital during the COVID-19 pandemic. The researcher stopped recruitment when sufficient information had been collected and no new information was likely to be added. The use of manifest qualitative content analysis is an established, conventional, and frequently used method in qualitative research [12]. The geographical spread of the patients recruited further increases external validity.

The interviews were only performed in Swedish, which excluded participation of non-Swedish-speaking patients. Further, those who could not engage with a computer or the internet were excluded, and this might have led to selection of patients who were more accustomed to information technology.

### 4.2 Implications for future research

In order to continually improve upon the design of clinical trials, it is important to consider the patient experience. When designing studies, patient perspectives are being increasingly considered, both directly with patients involved in the set up and design of a trial [18,19] and indirectly via research focusing on patient perspectives. This paper adds the perspectives of patients who are involved in a registry-based randomized trial using a technical product to improve adherence of study drug. In a population consisting of patients with myocardial infarction who may be under significant stress from their acute illness and therefore have difficulty remembering information, a registry-based approach is a feasible way to conduct research. A registry-based trial embedded in follow-up healthcare is also advantageous to patients because trial visits can be carried out within routine follow-up visits, thereby reducing the required time and travel commitments of recruited patients. However, the results of this study highlight that, when recruiting patients, it is important to have a person-centred approach. Individual information and partnership with patients are of great importance owing to their vulnerable condition. The smart cap has the possibility of being used to improve adherence to both study medication and other medications in different settings. The interviewee responses demonstrate that improvements in design to accommodate for patients with visual impairment and frailty would be useful.

## 5 Conclusions

Most patients in this interview study had a positive attitude to contributing to a clinical trial despite their acute medical condition. Above all, they appreciated verbal information and expressed great confidence in the research staff. The vulnerable condition in the acute setting was mostly evidenced by insufficient memory during the hospitalization period for acute myocardial infarction and difficulties coping with the diagnosis. The new use of a smart bottle cap for remote assessment of study drug adherence was well accepted by the patients.

## Funding

The DAPA-MI trial was funded by AstraZeneca. The conduct of the research presented in this paper was not funded by any specific grant from any funding agency in the public, commercial, or not-for-profit sectors. Funding for editorial assistance and article fees was provided by AstraZeneca.

## Research data for this article

Owing to the sensitive nature of questions asked in this study, full interview transcripts will not be shared to maintain patient confidentiality.

## CRediT authorship contribution statement

**Catrin Henriksson:** Writing – review & editing, Writing – original draft, Visualization, Methodology, Investigation, Formal analysis, Data curation, Conceptualization. **Anneli Olsson:** Writing – review & editing, Writing – original draft, Visualization, Methodology, Investigation, Formal analysis, Data curation, Conceptualization. **Kasper Andersen:** Writing – review & editing, Conceptualization. **Gabriel Arefalk:** Writing – review & editing, Conceptualization. **David Erlinge:** Writing – review & editing, Methodology, Conceptualization. **Robin Hofmann:** Writing – review & editing, Conceptualization. **Wilhelm Ridderstråle:** Writing – review & editing, Methodology, Conceptualization. **Annika Rutgersson:** Writing – review & editing, Methodology, Conceptualization. **Jonas Oldgren:** Writing – review & editing, Methodology, Conceptualization. **Stefan James:** Writing – review & editing, Methodology, Conceptualization.

## Declaration of competing interest

The authors declare the following financial interests/personal relationships which may be considered as potential competing interests: Wilhelm Ridderstråle and Annika Rutgersson are employees of AstraZeneca. All other authors declare no conflicts of interest.
